# An Economical Substitute for Fresh Milk

**Published:** 1919-09-27

**Authors:** 


### THE LATEST VIEWS ON VITAMINES.

### An Economical Substitute for Fresh Milk.

WITH the establishment by numerous investigations of the paramount importance of those ingredients of diet which are known as vitamines (and especially so in the case of the young, both of the human species and of animals) has come as a natural consequence a critical attitude towards many articles of infant and adult dietary which have hitherto been taken on trust. In a recently-published research, or series of researches, conducted at the Lister Institute by Dr. Harriette Chick and other workers, and published in the *Lancet*,* some very important new facts are disclosed concerning the vitamine content of various common articles of diet. And the application of this work, and of other recent researches along the same lines, is discussed in the same journal by Dr. E. A. Barton, medical officer to the infant department of University College Hospital,† who touches also on the economic aspects of the results of these researches.

Those who have followed the progress of recent medical thought on vitamines are aware that these peculiar substances, present only in the most minute quantities in fresh animal and vegetable foods, are of varying natures, as well as of varying quantity, in different foodstuffs. Some of the most important, and it is these that Drs. Chick, Barnes, Hume, Skelton, and Campbell have been investigating, are those which ward off scurvy: their total absence, or relative insufficiency, in the diet of man or animals is followed, sooner or later, by the symptoms, more or less severe (and not always easily recognisable in mild cases), of that peculiar disease. The conclusions reached appear, on the evidence offered, to be valid, though, of course, the data, as well as the inferences founded on them, remain to be confirmed by independent workers.

### VITAMINES IN CANNED VEGETABLES.

To begin with, some of the results of "canning" vegetables, from the point of view of vitamine content, have occupied attention. It is not surprising to learn that the greater part of the original anti-scorbutic properties of the raw vegetable is destroyed in this proceeding; indeed, the strange part is that any anti-scorbutic vitamine remains at all. In the case of runner-bean pods the loss is estimated at 90 per cent. of the original value; in the case of cabbage at 70 per cent. This loss is primarily due to the heating involved in the process of canning; and a further loss may be expected to occur during the period of storage. In the case of green-leaf vegetables, which possess, in addition to the anti-scurvy vitamine, the "fat-soluble" growth-promoting accessory factor, the latter substance is also lacking in the canned material unless the liquor be also taken. It is strongly emphasised that the value of canned vegetables, as regards anti-scorbutic and growth-promoting properties, must be regarded as negligible.

Of great importance also to those responsible for famine areas of India, and other officials similarly employed, are some researches on dry tamarind, cocum, and mango, which have been thought by distinguished officers of the Indian Medical Service to be efficient anti-scorbutics for troops in the field on the Indian borders. These three dried products are shown to have a small, but definite, anti-scorbutic value. This value is found to be greatly inferior to that of raw cabbage, swede, germinated pulses, orange juice, or lemon juice; but superior to that of carrots, beetroot, cooked potatoes, or raw meat juice, reckoned weight for weight in the natural condition. The low value attached to raw meat juice conflicts with the experience of recent Polar expeditions, where seal meat (cooked, or partly cooked) was found in actual experience to prevent, and cure, scurvy better than any other substance. Possibly the *caveat* entered by Dr. Barton, to be noticed presently, may apply also to these researches, which were carried out on animals, and may not apply in their entirety to human beings.

### THE RELATIVE EFFECTS OF FRESH, HEATED, AND DRIED MILKS.

Strange to say, raw milk (of the cow) has been found not to be in the first flight of foodstuffs as regards anti-scorbutic qualities, though it has, of course, very definite properties of that kind. It would be interesting to know whether this statement holds good for calves, as well as for the smaller laboratory animals on which the experiments have usually been tried; for it may be that the milk of any animal contains ample anti-scorbutic vitamines for its own young, but less for the young of other species. As might be expected, it is found that "scalded" milk is inferior to fresh milk in anti-scorbutic vitamine content, and that dried milk is inferior to both. These facts, as the authors quoted observe, form a strong argument for adding an extra anti-scorbutic to the diet of infants nourished on dried milk. The additions recommended are raw orange juice, raw swede juice, and juice of tomatoes, fresh or canned. Grape juice and carrot juice are also good, but not so potent as those previously mentioned. Winter milk is thought to be inferior to summer milk in anti-scorbutic action, owing to differences in the diet of cows at these seasons. Swedes are pronounced to be much superior to mangolds in this respect as winter diet for cows.

Curiously, no significant differences were detected in the growth-promoting properties of raw and dried milk respectively, from which the conclusion may possibly be allowable that dried milk

* August 23, 1919, page 320.
† August 23, 1919, page 348.

**The Latest Views on Vitamines—(*continued*).**

is permissible as an infant food, if care be taken to supply sufficient anti-scorbutic vitamines in another form.

### Bearing on Infant Welfare Work.

The application of these views to infant feeding and to the teaching disseminated through the community by infant welfare centres is obviously of the greatest importance. Yet, as Dr. Barton points out, it may be that the results of research in this matter do not apply so completely to the human young as to that of animals. It has been shown during the War that the absence or destruction of the anti-scorbutic element in dried milk has no apparent influence on growth, nor did Barlow's disease (infantile scurvy, so-called scurvy rickets) appear, save in very exceptional cases. Animals suffer from deprivation of the anti-scorbutic vitamine much more rapidly and more severely than human babies. The reason for this is not yet known, though it may be hoped that future research may reveal it.

Another interesting point raised is that possibly frank scurvy is the last chapter of a disease whose early manifestations are not recognised, but may possibly include defective dentition. At University College Hospital they take no chances, but add the juice of minced oranges, rind included, to the diet of artificially reared babies. The rind is used because it contains essential oils which preserve the juice for quite long periods—a small point, but one worth attending to.

### Artificial "Cream."

The fat-soluble factor is present in dried milk in sufficient quantity to prevent rickets, as has been mentioned. When the infant will not tolerate dried milk, and diluted cow's milk has to be substituted, the dilution may become an anxiety unless cream is added to the food. Cream nowadays is prohibitive in price for the poor, so the infant department at University College Hospital has evolved an artificial substitute which is rich in the fat-soluble vitamine, cannot go bad, and is cheap. The following is the recipe for this artificial cream: Beef suet, 40 oz.; olive oil, 5 oz.; syrup, 25 fl. oz.; benzoic acid, 35 gr.; decoction of Irish moss, 70 fl. oz.; water to one gallon. The oil is added to the melted suet and the benzoic acid dissolved in the mixture. The decoction is heated to about 50 degrees and placed in an emulsifier, and the fats are then added at about the same temperature. The emulsion is then worked up, and the syrup and water are added last—a method well worthy of the attention of those in charge of infant welfare centres who wish to do their best in the light of the most modern research for their little patients, and are faced by the difficulty of getting cream either pure or cheap.

